# 

**Published:** 1889-07

**Authors:** 


CONTENTS.

VOL. 36 No. 7.

| | PAGE. |
|---|---|
| Health Resorts | 145 |
| Health and Hell | 146 |
| Health without Medicine | 150 |
| Haste and Health | 154 |
| What Drags the Life Out of a Woman | 155 |
| The Jak Tree | 156 |
| Prevalence of Suicide | 158 |
| A Girl's Toilet Articles | 160 |
| Effect of Occupation, on the face | 160 |
| Ventilation | 161 |
| The Corset, a Cause of Consumption | 162 |
| Modern Indulgences | 162 |
| A Good Thing for Boys | 163 |
| How Celluloid is made | 164 |
| Book Notices | 165 |
| Household | 166 |
| Miscellaneous | 167 |
| A Ride | 168 |

INDEX TO ADVERTISEMENTS OF HALF A PAGE AND OVER.

| | PAGE. |
|---|---|
| Esoteric Publishing Co | I |
| Celluloid | II |
| Hollow Suppositories | III |
| E. C. Morris & Co | IV |
| Lactopeptine | V |
| Revised Premium List | VII |
| The Herb Cure Co | IX |
| Ladies' Home Companion | X |

